# Coronary artery calcium scoring in 2026: strengths, limitations, and optimized clinical use

**DOI:** 10.3389/fradi.2026.1822303

**Published:** 2026-04-13

**Authors:** Pierre Sabouret, Domenico Mario Giamundo, Julien Rosencher, Stefano Figliozzi

**Affiliations:** 1 Heart Institute and Action Group, Pitié-Salpétrière, Sorbonne University, Paris, France; 2 Department of Cardiology, Policlinico Casilino, Rome, Italy; 3 Department of Cardiology, CMC Ambroise Paré, Neuilly-sur-Seine, France; 4 Cardio Center, IRCCS Humanitas Research Hospital, Rozzano, Milan, Italy; 5 Department of Biomedical Sciences, Humanitas University, Pieve Emanuele, Milano, Italy

**Keywords:** atherosclerotic burden, cardiovascular risk stratification, coronary artery calcium (CAC) score, coronary CT imaging, primary prevention

## Abstract

Coronary artery calcium (CAC) scoring on non-contrast ECG-gated CT remains a robust, reproducible marker of total coronary atherosclerotic burden with clear prognostic value and consistent risk reclassification beyond contemporary clinical calculators. Recent studies (2018–2026) reinforce the ‘power of zero’ for near-term risk de-escalation, identify very high CAC (≥1,000) as a distinct, very-high-risk phenotype, and, importantly, provide randomized evidence that CAC-guided treatment reduces plaque progression. Advances in artificial intelligence (AI), spectral CT, and standardized reporting (SCCT/STR; CAC-DRS) expand opportunities for automated and incidental CAC detection. This mini-review summarizes updated strengths, limitations, and practice guidance; synthesizes new evidence (2024–2026); and includes a practical clinical decision flowchart for the selective use of CAC in prevention pathways.

## 1 Concept and prognostic power

CAC quantifies calcified plaque burden (Agatston score) and tracks cumulative atherosclerosis. Across the Multi-Ethnic Study of Atherosclerosis (MESA), CAC shows a graded association with 10-year ASCVD events across age, sex, and race/ethnicity (1); scores >100 universally indicate ≥7.5% 10-year risk (1), whereas CAC=0 conveys a prolonged ‘warranty’ period with very low event rates (2, 3). Very high CAC (≥1,000) identifies patients with event rates approaching treated secondary-prevention cohorts (4).

## 2 Key strengths

- Continuous risk gradient with superior discrimination and net reclassification in borderline/intermediate-risk adults (1).
- ‘Power of zero’: CAC=0 supports risk de-escalation and statin deferral in carefully selected patients without strong risk enhancers (2, 3).
- Patient engagement: visualizing calcification improves adherence and lifestyle change (5).
- Standardization: SCCT/STR guidance and CAC-DRS harmonize acquisition, reporting, and management language; incidental CAC on chest CT can be reported consistently (6–8).

## 3 Limitations and pitfalls

- Lack (until recently) of outcomes-driven RCTs testing CAC-guided strategies vs. guideline-directed care; population-screening trials altered treatment allocation but did not yet reduce all-cause mortality at early follow-up (9).
- Not a rule-out for obstructive CAD—especially in younger symptomatic patients where non-calcified plaque may be present despite CAC=0; use CCTA/functional testing when clinical suspicion is high (10, 11).
- Biologic surrogate: therapies can increase calcium density while stabilizing plaque, decoupling serial CAC change from short-term risk reduction—routine serial CAC to monitor therapy is not recommended (10, 11).
- Radiation/access and protocol heterogeneity persist without standardization; equity concerns if CAC is used only in well-resourced populations (7).
- CAC does not detect non-calcified plaque and may therefore underestimate risk in selected patients, particularly younger individuals or symptomatic patients with early-stage atherosclerosis.
- Widespread CAC implementation may also increase downstream testing and resource use if applied outside appropriately selected populations.

## 4 New evidence and practical takeaways

### 4.1 PREVENT+CAC

Adding CAC to PREVENT-based risk assessment improves risk discrimination and reclassification, supporting CAC as a useful imaging adjunct to contemporary risk models in selected patients (12).

### 4.2 CAC vs. polygenic risk

In MESA and the Rotterdam Study, CAC showed better discrimination than polygenic risk scores for prediction of coronary heart disease events and provided greater incremental value beyond traditional risk factors (13).

### 4.3 Trajectories and progression

Ten-year MESA analyses show distinct CAC trajectories with graded event risk; validated nomograms can help identify individuals likely to progress, informing timing for repeat CAC and intensification of prevention (1).

### 4.4 Randomized evidence (CAUGHT-CAD)

The CAUGHT-CAD trial provides randomized evidence that CAC-guided treatment intensification may influence plaque progression; however, its population consisted of patients with familial/hereditary coronary artery disease, and therefore generalizability to unselected primary prevention populations remains uncertain (14).

### 4.5 Imaging-guided outcomes (SCOT-HEART, 10 years)

CCTA-guided care (with routine CAC quantification) reduces myocardial infarction and MACE vs. standard care over a decade, supporting earlier anatomic imaging in stable chest pain pathways (8).

### 4.6 AI and spectral CT

AI-based approaches enable rapid CAC quantification from coronary CTA, while standardized assessment on noncardiac CT may facilitate opportunistic detection and prevention pathways (15, 16). Early studies suggest that dual-layer spectral CT with virtual non-contrast reconstruction, including deep-learning approaches, may allow CAC estimation without a dedicated non-contrast acquisition; however, further clinical validation is still needed (17, 18).

## 5 Clinical implications of the new studies

- Risk tools: Combine PREVENT with CAC to refine decisions in borderline/intermediate-risk adults; CAC can inform shared decision-making, particularly in borderline- or intermediate-risk individuals (12).
- Therapy thresholds: CAC >0 (especially ≥100) supports statin initiation/intensification; CAC ≥1,000 warrants aggressive LDL-C lowering (e.g., add ezetimibe/PCSK9i) and closer follow-up (4, 10, 11). However, although CAC ≥ 1,000 identifies a very-high-risk phenotype with event rates approaching those observed in secondary prevention populations, optimal management in this group remains uncertain. In particular, there is currently no dedicated randomized evidence to define LDL-C or glycemic targets, or to clarify the role of newer therapies such as PCSK9 inhibitors, bempedoic acid, or GLP-1 receptor agonists in this setting.
- Repeat CAC: Consider in selected patients guided by trajectory/progression models and evolving risk, rather than fixed intervals for all (1).
- Symptomatic pathways: Do not use CAC to rule out obstructive CAD; use CCTA or functional testing when symptoms merit (10, 11).
- Operationalization: Leverage SCCT/STR protocols and CAC-DRS, and implement automated incidental CAC reporting; AI workflows can scale prevention and reduce disparities (7, 8, 15, 16).
- Resource stewardship: CAC-first triage strategies may reduce unnecessary advanced testing (e.g., PET) and direct resources to those most likely to benefit.

These findings should be interpreted as proof-of-concept rather than definitive evidence for broad CAC-guided treatment implementation in the general borderline/intermediate-risk population.

## 6 Clinical decision flowchart (CAC-guided prevention)

**Figure 1:**
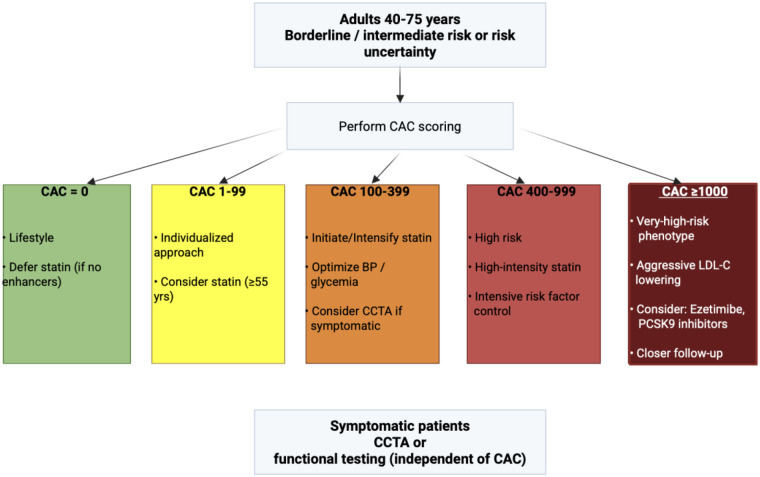
CAC-guided prevention pathway. CAC=0 → lifestyle and defer statin in absence of strong enhancers; CAC 1–99 → individualized, consider statin (especially ≥55 years); CAC 100–399 → initiate/intensify statin, optimize BP/glycemia, consider CCTA if symptomatic; CAC 400-999 → treat as high risk with intensive risk factor control; CAC ≥1,000 → consistent with a very-high-risk phenotype, with consideration of aggressive LDL-C lowering and additional lipid-lowering therapies (e.g., ezetimibe or PCSK9 inhibitors); symptomatic patients require anatomic/functional testing irrespective of CAC.

Figure 1 embeds a pragmatic prevention pathway for adults 40–75 years with borderline/intermediate 10-year risk or risk uncertainty. It integrates PREVENT + CAC, randomized data on plaque progression (CAUGHT-CAD), and long-term CCTA outcomes (SCOT-HEART).

## References

[1] Budoff MJ Young R Burke G Jeffrey Carr J Detrano RC Folsom AR Ten-year association of coronary artery calcium with atherosclerotic cardiovascular disease (ASCVD) events: the multi-ethnic study of atherosclerosis (MESA). Eur Heart J. (2018) 39(25):2401–8. 10.1093/eurheartj/ehy217 29688297 PMC6030975

[2] Dzaye O Dardari ZA Cainzos-Achirica M Blankstein R Agatston AS Duebgen M Warranty period of a calcium score of zero: comprehensive analysis from MESA. JACC Cardiovasc Imaging. (2021) 14(5):990–1002. 10.1016/j.jcmg.2020.06.048 33129734 PMC8076346

[3] Valenti V Ó Hartaigh B Heo R Cho I Schulman-Marcus J Gransar H A 15-year warranty period for asymptomatic individuals without coronary artery calcium: a prospective follow-up of 9,715 individuals. JACC Cardiovasc Imaging. (2015) 8(8):900–9. 10.1016/j.jcmg.2015.01.025 26189116 PMC4537357

[4] Peng AW Dardari ZA Blumenthal RS Dzaye O Obisesan OH Iftekhar Uddin SM Very high coronary artery calcium (≥1000) and association with cardiovascular disease events, non-cardiovascular disease outcomes, and mortality: results from MESA. Circulation. (2021) 143(16):1571–83. 10.1161/CIRCULATIONAHA.120.050545 33650435 PMC8058297

[5] Rozanski A Gransar H Shaw LJ Kim J Miranda-Peats L Wong ND Impact of coronary artery calcium scanning on coronary risk factors and downstream testing the EISNER (Early Identification of Subclinical Atherosclerosis by Noninvasive Imaging Research) prospective randomized trial. J Am Coll Cardiol. (2011) 57(15):1622–32. 10.1016/j.jacc.2011.01.019 21439754 PMC3104928

[6] Hecht HS Cronin P Blaha MJ Budoff MJ Kazerooni EA Narula J 2016 SCCT/STR guidelines for coronary artery calcium scoring of noncontrast noncardiac chest CT scans: a report of the Society of Cardiovascular Computed Tomography and Society of Thoracic Radiology. J Cardiovasc Comput Tomogr. (2017) 11(1):74–84. 10.1016/j.jcct.2016.11.003 27916431

[7] Hecht HS Blaha MJ Kazerooni EA Cury RC Budoff M Leipsic J CAC-DRS: coronary artery calcium data and reporting system. An expert consensus document of the Society of Cardiovascular Computed Tomography (SCCT). J Cardiovasc Comput Tomogr. (2018) 12(3):185–91. 10.1016/j.jcct.2018.03.008 29793848

[8] Williams MC Wereski R Tuck C Adamson PD Shah ASV van Beek EJR Coronary CT angiography-guided management of patients with stable chest pain: 10-year outcomes from the SCOT-HEART randomised controlled trial in Scotland. Lancet. (2025) 405(10475):329–37. 10.1016/S0140-6736(24)02679-5 39863372

[9] Lindholt JS Søgaard R Rasmussen LM Mejldal A Lambrechtsen J Steffensen FH Five-year outcomes of the Danish Cardiovascular Screening (DANCAVAS) trial. N Engl J Med. (2022) 387(15):1385–94. 10.1056/NEJMoa2208681 36027560

[10] Arnett DK Blumenthal RS Albert MA Buroker AB Goldberger ZD Hahn EJ 2019 ACC/AHA guideline on the primary prevention of cardiovascular disease: a report of the American College of Cardiology/American Heart Association Task Force on Clinical Practice Guidelines. Circulation. (2019) 140(11):e596–646. 10.1161/CIR.0000000000000678 30879355 PMC7734661

[11] Visseren FLJ Mach F Smulders YM Carballo D Koskinas KC Bäck M 2021 ESC guidelines on cardiovascular disease prevention in clinical practice. Eur Heart J. (2021) 42(34):3227–337. 10.1093/eurheartj/ehab484 34458905

[12] Rhee AJ Pandit K Berger JS Iturrate E Coresh J Khan SS Real-world evidence linking the predicting risk of cardiovascular disease events risk score and coronary artery calcium. J Am Heart Assoc. (2025) 14(11):e038991. 10.1161/JAHA.124.038991 40396415 PMC12229192

[13] Khan SS Post WS Guo X Tan J Zhu F Bos D Coronary artery calcium score and polygenic risk score for the prediction of coronary heart disease events. JAMA. (2023) 329(20):1768–77. 10.1001/jama.2023.7575 37219552 PMC10208141

[14] Nerlekar N Vasanthakumar SA Whitmore K Soh CH Chan J Goel V Coronary artery calcium score: use to guide management of hereditary coronary artery disease (CAUGHT-CAD) investigators. Effects of combining coronary calcium score with treatment on plaque progression in familial coronary artery disease: a randomized clinical trial. JAMA. (2025) 333(16):1403–12. 10.1001/jama.2025.0584 40042839 PMC11883595

[15] Dzaye O Razavi AC Jelwan YA Peng AW Grant JK Blaha MJ. Coronary artery calcium scoring on dedicated cardiac CT and noncardiac CT scans. Radiol Cardiothorac Imaging. (2025) 7(5):e240548. 10.1148/ryct.240548 40965299 PMC12583114

[16] Mu D Bai J Chen W Yu H Liang J Yin K Calcium scoring at coronary CT angiography using deep learning. Radiology. (2022) 303(2):306–14. 10.1148/radiol.2021211483 34812674

[17] Vargas EE Koetzier LR Tetteroo PM Langzam E Greuter MJW Velthuis BK A novel deep-learning approach for coronary artery calcium scoring in contrast-enhanced spectral coronary CT angiography: a phantom study. Eur J Radiol. (2026) 194:112479. 10.1016/j.ejrad.2025.112479

[18] Langenbach IL Wienemann H Klein K Scholtz JE Pennig L Langzam E Coronary calcium scoring using virtual non-contrast reconstructions on a dual-layer spectral CT system: feasibility in the clinical practice. Eur J Radiol. (2023) 158:110681. 10.1016/j.ejrad.2022.110681 36592582

